# New pathogenic variants of ALMS1 gene in two Chinese families with Alström Syndrome

**DOI:** 10.1186/s12886-022-02597-3

**Published:** 2022-09-26

**Authors:** Wan-Yu Cheng, Mei-Jiao Ma, Shi-Qin Yuan, Xiao-long Qi, Wei-Ning Rong, Xun-Lun Sheng

**Affiliations:** 1 Third Clinical Medical College of Ningxia Medical University, Ningxia Eye Hospital, People’s Hospital of Ningxia Hui Autonomous Region, No 936, Huanghe East Road, Jinfeng District, Yinchuan, 750001 Ningxia China; 2 Gansu Aier Ophthalmology and Optometry Hospital, 1228-437, Guazhou Road, Qilihe District, Lanzhou City, 730050 Gansu China

**Keywords:** Alström Syndrome, ALMS1, Gene mutation, Clinical manifestation, Pathogenicity

## Abstract

**Purpose:**

Alström Syndrome (AS) is an autosomal recessive hereditary disease with the characteristics of multiorgan dysfunction. Due to the heterogeneity of clinical manifestations of AS, genetic testing is crucial for the diagnosis of AS. Herein, we used whole-exome sequencing (WES) to determine the genetic causes and characterize the clinical features of three affected patients in two Chinese families with Alström Syndrome.

**Materials and methods:**

Three affected patients (initially diagnosed as achromatopsia). and five asymptomatic members were recruited for both genetic and clinical tests. The complete ophthalmic examinations and systemic examinations were performed on all participants. Whole exome sequencing (WES) was performed for mutation detection. The silico analysis was also applied to predict the pathogenesis of identified pathogenic variants.

**Results:**

In family 1, the proband showed low vision, hyperopia, photophobia, nystagmus, and total color blindness. DNA analysis revealed that she carried a compound heterozygote with two novel pathogenic variants in the *ALMS1* gene NM_015120.4:c.10379del (NP_055935.4:p.(Asp2252Tyr)) and NM_015120.4:c.11641_11642del (NP_055935.4:p.(Val3881ThrfsTer11)). Further systemic examinations showed short stature, acanthosis nigricans, and sensorineural hearing loss. In family 2, two affected siblings presented the low vision, hyperopia, photophobia, nystagmus, and total color blindness. DNA analysis revealed that they carried a same compound heterozygote with two novel pathogenic variants in the *ALMS1* gene NM_015120.4:c.10379del (NP_055935.4:p.(Asn3460IlefsTer49)), NM_015120.4:c.10819C > T (NP_055935.4:p.(Arg3607Trp)). Further systemic examinations showed obesity and mild abnormalities of lipid metabolism. According to the genetic testing results and further systemic analysis, the three affected patients were finally diagnosed as Alström Syndrome (AS).

**Conclusions:**

We found two new compound heterozygous pathogenic variants of the *ALMS1* gene and determined the diagnosis as Alström Syndrome in three patients of two Chinese families. Our study extends the genotypic and phenotypic spectrums for ALMS1 -AS and emphasizes the importance of gene testing in assisting the clinical diagnosis for cases with phenotypic diversities, which would help the AS patients with early diagnosis and treatment to reduce future systemic damage.

## Introduction

Alström Syndrome (OMIM 203,800, AS) is an autosomal recessive hereditary disease caused by structural alterations and dysfunction of primary cilia, characterized by multiorgan dysfunction and highly phenotypic heterogeneity [1, 2, 3]. Ciliopathies are defined as a group of genetically heterogeneous diseases caused by pathogenic variants in genes whose products are localized in the cilia-centrosome complex [4]. Cilia are widely distributed in the cell membrane of most eukaryotic cells and are involved in many signaling pathways in vivo, and cilia-mediated signals can affect the cell cycle [5]. *ALMS1* gene is the only gene identified to be associated with the pathogenesis of AS. ALMS1 is a ubiquitous protein that localizes to centrosomes and basal bodies of ciliated cells, which is widely expressed in the ciliary cell of the central nervous system, photoreceptors, endocrine systems, extracorporeal circulation system, and genitourinary system [6]. The protein encoded by the *ALMS1* gene has been implicated in ciliary function, cell cycle control, and intracellular transport. *ALMS1* gene function deficiency has been associated with primary cilia formation, localization, and dysfunction. However, the exact role of the ALMS1 protein remains uncertain, and there is no clear link with other ciliopathy proteins. Like many different genes, *ALMS1* expresses in several splice variants. Although the splicing patterns and functions of ALMS1 are not fully understood, previous studies have suggested roles for the protein in intracellular trafficking and ciliary function.

As a ciliary gene, *ALMS1* pathogenic variants can lead to a wide range of clinical features involving the eye, ear, kidney, heart, liver, central nervous system, adipose tissue, gonads, and bones [7, 8], including cone-rod dystrophy, hearing loss, type 2 diabetes, insulin resistance with hyperinsulinemia, dilated cardiomyopathy, and progressive hepatic, renal failure and hyperinsulinemia as the main clinical features [3]. Geberhiwot et al. [7] proved the hypothesis that adipose tissue dysfunction may be a key determinant of accelerated insulin resistance in Alström Syndrome cohort, through mouse experiment show that reduced *ALMS1* gene expression decreases adipogenesis and lipid accumulation. Although the clinical manifestations of AS vary significantly at different ages, the first ocular symptoms are vision loss, nystagmus, photophobia, which may be the first reason for children visiting ophthalmology clinic, and an important clue to the clinician's detection of underlying diseases.

Alström Syndrome is rare, with a prevalence of approximately 1:10,000–1,000,000 in the population [9], and only a few cases have been reported in China [10]. The pathogenesis of the disease is still unclear, treatment is mainly for symptomatic, with poor prognosis, and the average life expectancy of patients does not exceed 50 years. At present, early diagnosis and early comprehensive intervention can be carried out to delay the progression of the disease and prolong the life of patients, and improve the quality of life. However, due to the clinical heterogeneity phenotype of AS, it is difficult to determine the diagnosis of Alström Syndrome only by clinical examination. Genetic testing to identify the causative gene is necessary for AS diagnosis. Herein, we used whole-exome sequencing (WES) to determine the genetic causes and characterize the clinical features of three affected patients with initially clinically diagnosed as achromatopsia in two Chinese families with Alström Syndrome.

## Material and methods

### Patients and clinical evaluations

Our study, which adhered to the Declaration of Helsinki, was approved and reviewed by the Ethics Committee on Human Research at People Hospital of Ningxia Hui Autonomous Region. Before participation, written informed consent was received from each participant or their legal guardians. Three children with Alström Syndrome and their parents from two families were recruited for both genetic and clinical tests, family history was recorded, especially history of consanguineous marriage and history of preterm birth. Routine ophthalmic examinations were performed, including uncorrected visual acuities, best-corrected visual acuities (BCVAs), color vision tests, slit-lamp examination, color fundus photography (Heidelberg Engineering GmbH, Heidelberg, Germany), and optical coherence tomography (OCT) examinations (HD-OCT4000, Carl Zeiss Meditec, USA). And systemic examination, including serum liver and kidney function and electrolyte tests, hepatobiliary, pancreatic, spleen, bilateral renal portal ultrasound, and cardiac color Doppler ultrasound. Peripheral venous blood samples (5 mL) were collected from 3 patients and their parents for genomic DNA extraction.

### Genetic analysis

Samples of peripheral venous blood (5 ml) were collected from 3 patients and their parents in tubes containing ethylenediaminetetraacetic acid (EDTA); Genomic DNA was extracted using an automated DNA extractor (QIAGEN, Hilden, Germany). To reveal causing pathogenic variants in two families, the whole-exome sequencing approach was selectively performed on two families. Briefly, the libraries for the whole-exome sequence were established from the DNA samples using an exon capture kit (SureSelect ver. 6 + UTR, Agilent Technologies), according to the manufacturer’s instructions. The exons were sequenced as 100-basepairs paired-end reads by an Illumina HiSeq2500 (Illumina). After sequencing, the raw data were saved in FASTQ format, followed by the bioinformatics analysis. The data would be transformed to VCF format, variants were further annotated by ANNOVAR (http://annovar.openbioinformatics.org/en/latest/) and associated with multiple databases, such as 1000 genome (https://www.internationalgenome.org/), dbSNP (https://www.ncbi.nlm.nih.gov/snp/), HGMD (http://www.biobase-international.com/product/hgmd), and confirmed associated pathogenic gene mutational site by PCR and Sanger sequencing.

### In silico* analysis*

Publicly available servers for bioinformatic prediction tools such as Mutation Taster (http://www.mutationtaster), Combined Annotation Dependent Depletion, CADD (http://cadd.gs.washington.edu/snv) were used to predict the effect of two novel mutation sites of the Alström syndrome associated pathogenic gene (*ALMS1*) on its protein function. The scores predicted results A: Disease-causing automatic (harmful), D: Disease-causing (possibly harmful), N: Polymorphism (probably harmless), P: Polymorphism automatic (harmless), where Disease-causing automatic and Polymorphism automatic indicate that the variant is documented in available databases with clear evidence. The scores of CADD analysis are between 0 and 1, disease probability is higher as the score nears 1. 1.4 *Literature review.*

A search was conducted on PubMed using " Alström Syndrome,” "ALMS1″, "Mutation” "exon,” "ciliopathy" as the term to search the relevant literature between 2004 and 2020 to analyze the relationship between Alström Syndrome genotypes and clinical phenotypes.

## Results

### Clinical manifestation

Clinical features and identified pathogenic gene pathogenic variants of two families enrolled in this study are summarized in Table 1. The three patients were full-term births, non-inbreeding marriage of parents, no family history for an inherited disease. All three patients had the common clinical features including severe visual impairment, high hyperopia refractive errors, photophobia, nystagmus, total color blindness, and obesity.

**Table 1:** Clinical features in three patients from two families with Alström Syndrome

Family	Patient	Sex	Age	Refraction (OD)	Refraction (OS)	Ocular findings	BMI	Others
Family1	II:1	F	8	+ 7.50DS	+ 8.00DS	Photophobia Nystagmus Total color blindness	17.6	Obesity NHL Acanthosis nigricans
Family2	II:1	M	6	+ 7.25DS/ + 1.75DC*75°	+ 7.50DS/ + 1.25DC*85°	Photophobia Nystagmus Total color blindness	22.7	Obesity Abnormal lipid metabolism
	II:2	M	3	+ 4.75DS/ + 1.25DC*10	4.75DS/ + 1.25DC*65°	Photophobia Nystagmus Total color blindness	21.9	Obesity

### Family1

**Fig.1:**
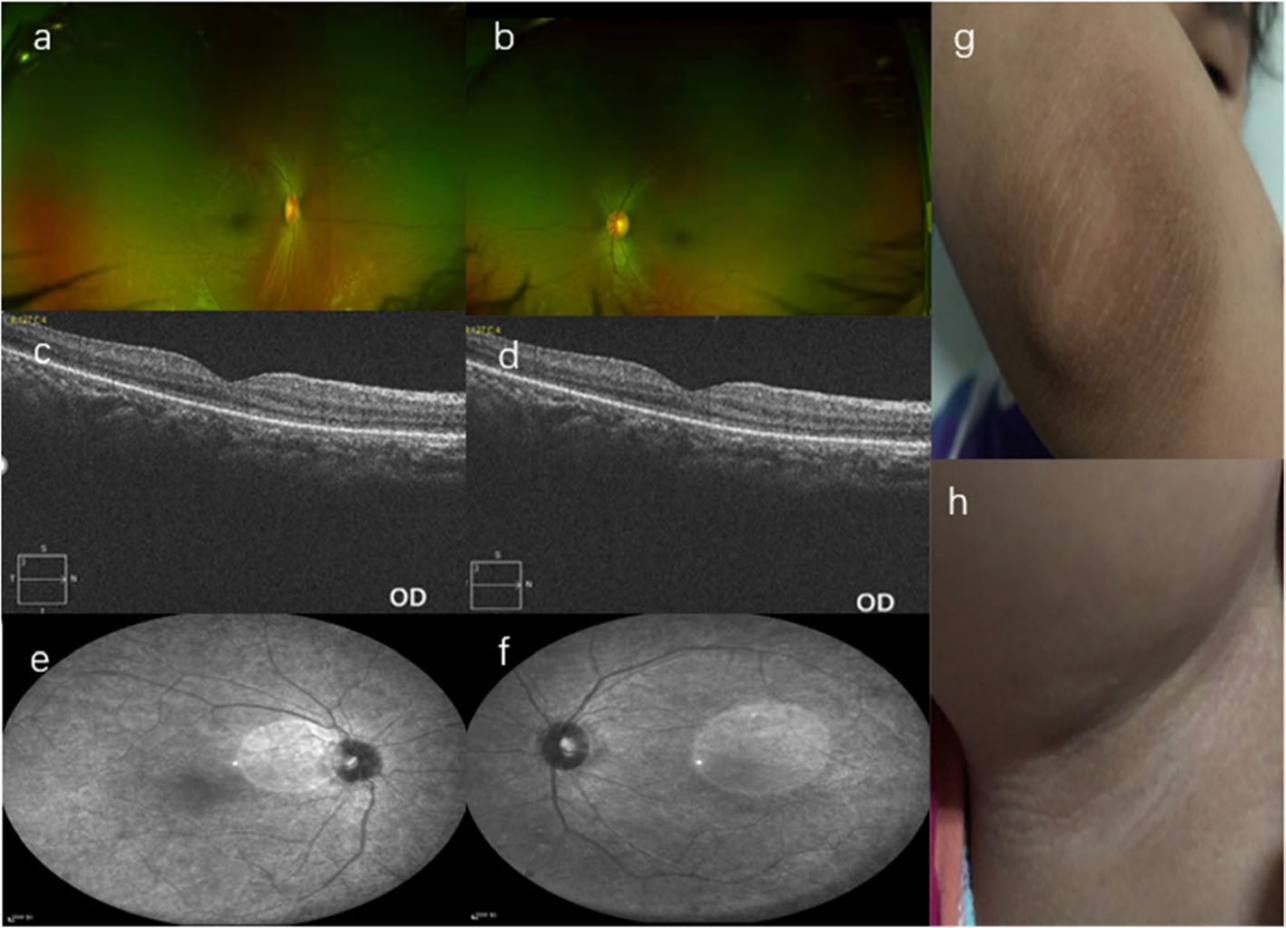
Fundus and body skin examination of the family 1 with Alström Syndrome. **a**, **b** Fundus photography of the individuals from II:1 showing nothing changed. **c**, **d** Optical Coherence tomography (OCT): showing standard foveal architecture in the II:1. **e**, **f** Fundus Autofluorescence (FAF): showing normal in the II:1. **g**, **h** Skin photography of the elbow and knee joint: showing acanthosis nigricans

The proband (II:1), an 8-year-old girl, had been initially referred for had poor visual acuity in both eyes. Uncorrected visual acuities were 0.01, and best-corrected visual acuities (BCVAs) were 0.03 on both eyes. The proband also showed photophobia, nystagmus and color vision loss. Fundus photography and OCT analysis showed normal (Fig. 1). ERG recording revealed mildly declined scotopic and severe reduced photopic responses. All of the examinations of the proband parents showed normal. According to the ocular phenotypes, the initial diagnosis was made as achromatopsia before genetic testing. The systemic manifestations of the proband included sensorineural hearing loss (SNHL) and acanthosis nigricans. Obesity can be diagnosed according to body mass index (BMI). The cardiac ultrasound examination did not find abnormal in both structure and function. Abdominal ultrasound uterine and adnexal ultrasound showed no abnormalities. No abnormalities were observed in serum liver and kidney function, glycosylated hemoglobin, thyroid function test, and sex hormones.

### Family2

**Fig. 2:**
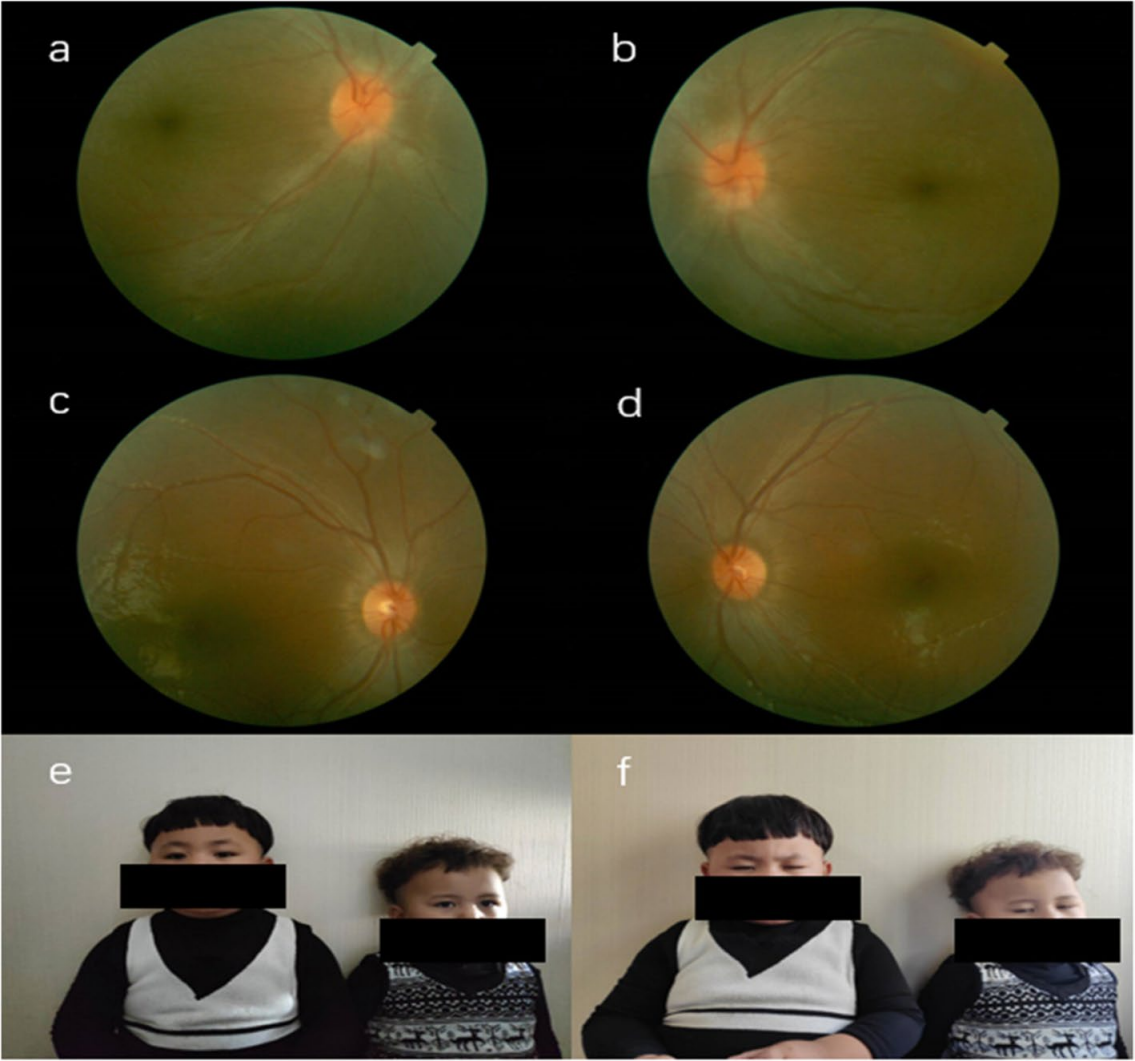
Photos of fundus, face, and figure of the two patients from family 2 with Alström Syndrome. **a**, **b** Fundus photographs from Family2 II:1: showing no abnormalities. **c**, **d** Fundus photographs from Family2 II:2: showing no abnormalities. **e**, **f** Figure photos of the body: showing obesity in two children. Face photo was taken in a low-light room, showing open eyes typically of both patients **e**. Face photo was born in a room with normal light: indicating photophobia of both patients **f**

The two patients (II:1 and II:2), presented with decreased visual acuity in both eyes. The best-corrected visual acuities (BCVAs) were CF/20 cm. Ophthalmologic examination showed photophobia nystagmus and total color blindness. No abnormality was found in anterior ocular segments and fundus in both children (Fig. 2). Both patients could not cooperate with ERG and OCT examination due to severe nystagmus. According to the ocular phenotypes, the initial diagnosis was made as achromatopsia before genetic testing. Hyperphagia are not exist with two patients, obesity can be diagnosed according to body mass index (BMI). Blood lipid examination found II:1 has abnormal lipid metabolism. The cardiac ultrasound examination did not find abnormal in both structure and function. Abdominal ultrasound uterine and adnexal ultrasound showed no abnormalities.

### Genetic findings

**Fig. 3:**
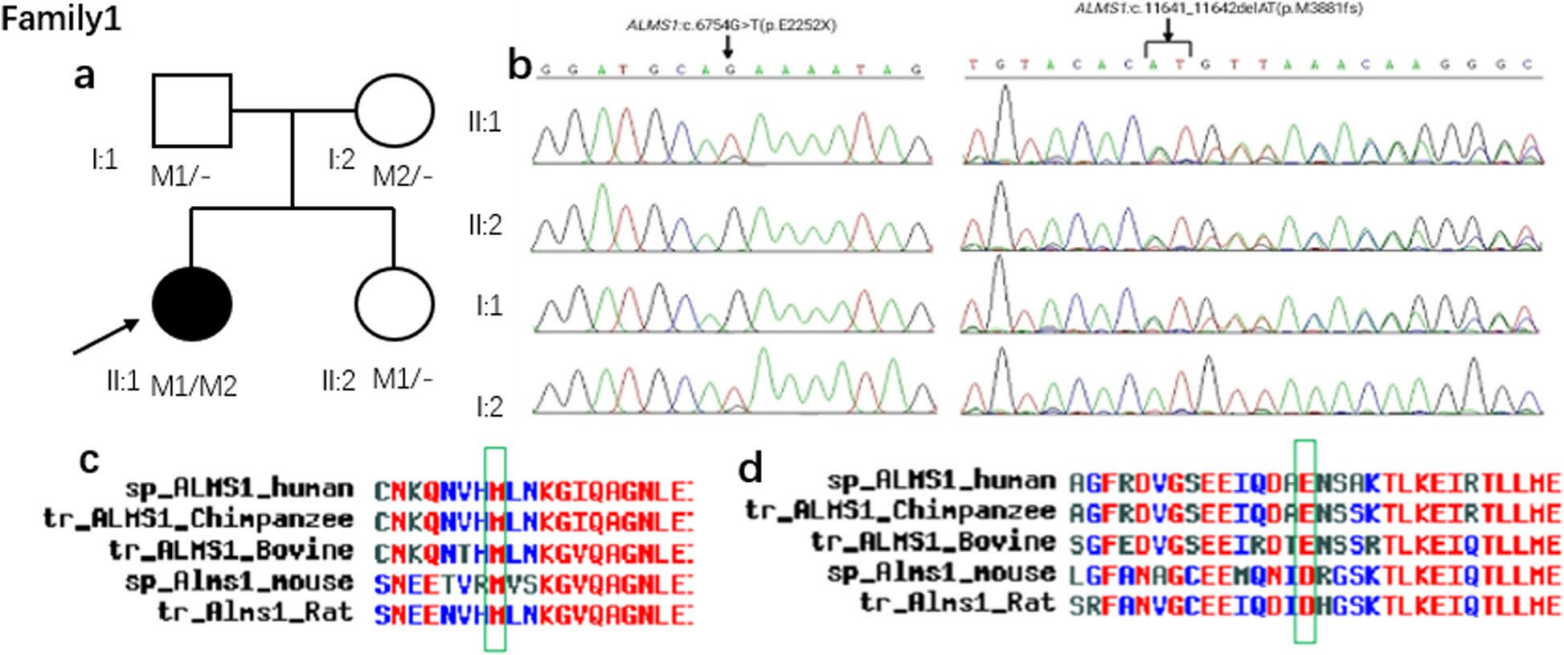
Sequence analysis and identification of the novel pathogenic variants of ALMS in the affected Chinese family 1 with Alström Syndrome. **a** Pedigree of the family. The filled black symbols represent the fake members, and the arrow denotes the proband. **b** By sequencing analysis, compound heterozygous variants of M1: NM_015120.4:c.11641_11642del (NP_055935.4:p.(Val3881ThrfsTer11)) and M2: NM_015120.4:c.6754G > T (NP_055935.4:p.(Asp2252Tyr)) were identified in the affected individuals of II:1. **c** The homology of amino acid sequences between human *ALMS1* and other species. The amino acid at position 3881 (Methionine, M3881) is highly conserved among species (**d**) The homology of amino acid sequences between human ALMS and other species. At position 2252 (Glutamic acid, E2252), the amino acid is relatively conserved among species

In family 1, using the whole-exome sequencing, we initially found compound heterozygous variants NM_015120.4:c.6754G>T（NP_055935.4:p.(Asp2252Tyr) and NM_015120.4:c.11641_11642del（p.M3881fs）of *ALMS1 *gene in the proband(II:1). And then segregated the disease status; these two variants were later confirmed in their parents (II:1 and I:2), younger sister (II:2) respectively, by Sanger sequencing (Figure 3). The heterozygous *ALMS1 *gene variant NM_015120.4:c.6754G>T（NP_055935.4:p.(Asp2252Tyr), located in exon 8, was found in her mother (I:2), and the variant NM_015120.4:c.11641_11642del（NP_055935.4:p.(Val3881ThrfsTer11), located in exon17, was found in her father(I:1) and proband younger sister (II:2).

**Fig. 4:**
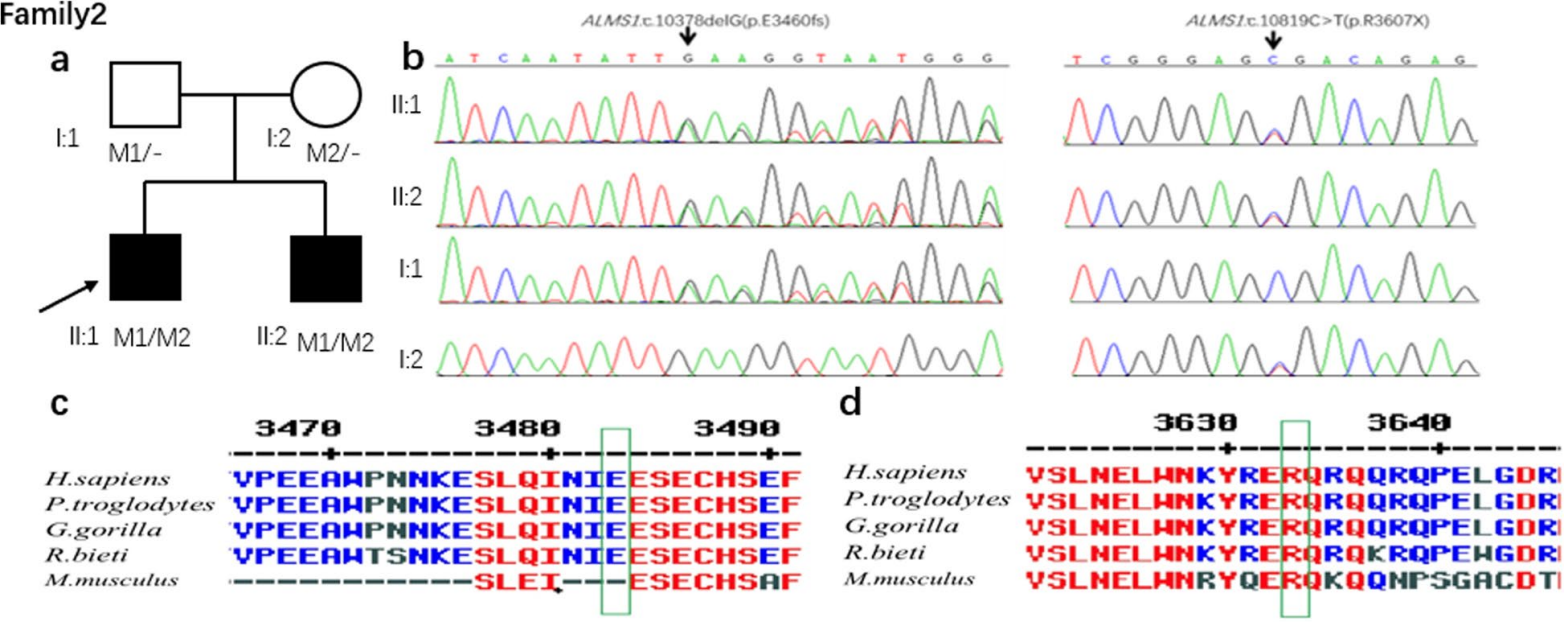
Sequence analysis and identification of the novel pathogenic variants of ALMS in the affected Chinese family 2 with Alström Syndrome. **a** Pedigree of the family. The filled black symbols represent the fake members, and the arrow denotes the proband. **b** By sequencing analysis, compound heterozygous variants of M1: NM_015120.4:c.10379del (NP_055935.4:p.(Asn3460IlefsTer49)) and M2: NM_015120.4:c.10819C > T (NP_055935.4:p.(Arg3607Trp)) were identified in the affected individuals of II:1 and II:2. **c** The homology of amino acid sequences between human ALMS and other species. The amino acid at position 3640 (Glutamic acid, E3460) is highly conserved among species **d** The homology of amino acid sequences between human ALMS and other species. The amino acid at position 3607 (Arginine, R3607) is highly conserved among species

In family 2, using the whole-exome sequencing, we found compound heterozygous variants NM_015120.4:c.10379del（NP_055935.4:p.(Asn3460IlefsTer49)）and NM_015120.4:c.10819C>T（NP_055935.4:p.(Arg3607Trp)）of* ALMS1 *gene in two affected siblings(II:1 and II:2), and then segregated the disease status; these two variants were later confirmed in their parents (I:1 and I:2) respectively, by Sanger sequencing (Figure 4). The heterozygous *ALMS1 *gene variant NM_015120.4:c.10379del（NP_055935.4:p.(Asn3460IlefsTer49), located in exon15, was found in their father(I:1), and variant NM_015120.4:c.10819C>T（NP_055935.4:p.(Arg3607Trp), located in exon16, was found in their mother(I:2). The detected pathogenic variants are summarized in Table 2.

**Table 2:** ALMS1 gene pathogenic variants identified in patients with Alström Syndrome

Family	Patient	Exon	Genotype	cDNA change	Protein change	variation source
Family1	II:1	Exon8	compound heterozygous	NM_015120.4:c.6754G > T^a^	NP_055935.4:p.(Asp2252Tyr)	mother
		Exon17		NM_015120.4:c.11641_11642del^a^	NP_055935.4:p.(Val3881ThrfsTer11)	father
Family2	II:1	Exon15	compound heterozygous	NM_015120.4:c.10379del^a^	NP_055935.4:p.(Asn3460IlefsTer49)	father
		Exon16		NM_015120.4:c.10819C > T^a^	NP_055935.4:p.(Arg3607Trp)	mother
	II:2	Exon15	compound heterozygous	NM_015120.4:c.10379del^a^	NP_055935.4:p.(Asn3460IlefsTer49)	father
		Exon16		NM_015120.4:c.10819C > T^a^	NP_055935.4:p.(Arg3607Trp)	mother

^a^New pathogenic variants

These four variants have not been previously reported in the literature and were also not seen in the ordinary people database of gene sequencing companies (Agilent Technologies, Inc). Searching for the 1000 genomes database (including 301 Chinese), and the exome aggregation consortium database (including all races, male: 33,644, female: 27,062, total: 60,706) mutational frequency of these four variants was equal to 0. Therefore, the pathogenicity of the four novel ALMS1 pathogenic variants in these two Chinese AS families, NM_015120.4:c.6754G > T, NM_015120.4:c.11641_11642del, NM_015120.4:c.10819C > T, and NM_015120.4:c.10379del were further predicted by subsequent analysis of the function of the relevant protein.

In family 1, the prediction programs performed the variant of NM_015120.4:c.6754G > T in silico analysis—predicted A&A&A with Mutation Taster indicating harmful. And obtained prediction score of 0.97008 by CADD_raw rank score indicating deleterious. (Table 3). According to ACMG [11] (Standards and Guidelines for the classification of genetic variation, specified by The American College of Medical Genetics and Genomics, ACMG). Nonsense and frameshift pathogenic variants are considered solid pathogenic evidence. By Sanger sequencing, comparing with genotype and clinical phenotype, the results are in accord with cosegregation analysis. Meanwhile, cosegregation analysis is seen as pathogenic support evidence. The amino acid at position 3881 (Methionine, M3881) and position 2252 (Glutamic acid, E2252) are conserved among species. Therefore, the compound heterozygous variant of family1 most likely induces pathopoiesia. In family 2, the prediction programs performed the variant of c.10819C > T in silico analysis. Predicted as A&A with Mutation Taster indicating harmful, and obtained prediction score of 0.99936 by CADD_raw_rankscore indicating deleterious (Table 3). Also, according to the ACMG, NP_055935.4:p.(Arg3607Trp)and NP_055935.4:p.(Asn3460IlefsTer49) pathogenic variants are considered solid pathogenic evidence. By Sanger sequencing, comparing with genotype and clinical phenotype, the results are in accord with cosegregation analysis. The amino acid at position 3640 (Glutamic acid, E3460) and the amino acid at position 3607 (Arginine, R3607) are highly conserved among species, indicating that pathogenic variants at these sites are more likely to affect the structure and function of ALMS1 protein.

**Table 3:** The effect of ALMS1 variants on their protein function by in silico analysis

Software	Variants	Score	Predicted signal
Mutation taster	NM_015120.4:c.6754G > T	A and A and A,	Disease-causing automatic
CADD_raw_rankscore	NM_015120.4:c.6754G > T	0.97008	damaging
Mutation taster	NM_015120.4:c.10819C > T	A and A	Disease-causing automatic
CADD_raw_rankscore	NM_015120.4:c.10819C > T	0.99936	damaging

To sum up, each of the two pathogenic variants is most likely the causative mutation for the disease phenotype in two families

### Some literature review

A search of the previously reported relevant literature showed that pathogenic variants in the *ALMS1* gene resulted in a complex and diverse ocular phenotype. The Ocular phenotype of most of the previously reported cases was vision loss, nystagmus, and photophobia starting in early childhood. Most of them were clinically diagnosed as cone-rod dystrophy (CORD).

ALMS1 protein has a role in maintaining cilia function and structure and plays a role in intracellular transport, ciliary signaling pathway regulation, and cell differentiation [12]. It is widely expressed in ciliary cell centrosomes, basal and cytosolic tissues of the central nervous system, photoreceptors, endocrine system, extracorporeal circulatory system, and urogenital system, the clinical manifestations of AS are diverse and have individual differences [13], as shown in Table 4.

**Table 4:** Clinical manifestations of systemic organ involvement caused by pathogenic variants in *ALMS1* gene

Literature	General manifestation						
	Obesity	Heart	Kidney	Diabetes	Hormone level	Hearing	Others
Torkamandi et al.[14]	+	Diastolic dilated cardiomyopathy	Renal ultrasound shows hyperechoic renal medulla	Type II diabetes	Hypertriglyceridemia, hyperlipidemia	Neurological hearing loss from age 4	Acanthosis nigricans
Etheridge T et al.[15]	+	Dilated cardiomyopathy	-	-	Elevated triglyceride	Mild hearing loss	Cryptorchidism, history of Burkitt's lymphoma, family history of consanguineous marriage
Nasser F et al.[16]	+	Myocarditis cardiomyopathy	-	Type II diabetes	Elevated transaminase level	Hearing loss	Inactive thyroid nodules, hypogonadism
Gatticchi et al.[17]	-	Decreased left ventricular function	Diapers are still needed up to age 3	-	Hypercholesterolemia and hypertriglyceridemia	Chronic cicatricial inflammation of the middle ear, hearing loss	Biopsy specimens from pharyngeal bronchoscopy showed reduced cilia number and attention deficit and hyperkinetic behavior;
Aldrees A, et al.[18]	+	Not mentioned	Not mentioned	Not mentioned	Not mentioned	Neurological hearing loss	Family history of consanguineous marriage
Dassie F et al.[19]	+	Cardiological disorders	Not mentioned	Type II diabetes	hepatic steatosis	mild bilateral sensorineural hearing loss	difficulties in the auditory working memory with ideomotor and buccofacial apraxia

## Discussion

This study revealed four novel pathogenic variants of *ALMS1* gene and analyzed the clinical phenotype of the three patients and the other members in two Chinese families with Alström Syndrome.

The *ALMS1* gene, located on Chromosome 2, consists of 23 exons and encodes a protein of 4169 amino acids [14, 20]. At present, five types of *ALMS1* pathogenic variants have been reported in the Human Gene Mutation Database (HGMD), mainly including frameshift pathogenic variants, nonsense pathogenic variants, missense pathogenic variants, etc. The most significant proportion of these pathogenic variants is frameshift pathogenic variants and nonsense pathogenic variants, almost half of which occur in exon 8, which may be related to the more extended exon 8 (6.1 kb, accounting for 49% of the coding sequence). A study by Marshall et al. [20], on the mutation spectrum of the *ALMS1* gene included a total of 239 previously reported cases of *ALMS1* pathogenic variants, with 357 mutant loci detected in 204 families (408 alleles) and a mutation detection rate of 88%. The most mutant loci were found in exon 8, accounting for 49% of the total, followed by exon 10 and exon 16.

In this study, we found two compound heterozygote with four novel pathogenic variants in the *ALMS1* gene NM_015120.4:c.6754G > T, NM_015120.4:c.11641_11642del, NM_015120.4:c.10819C > T and NM_015120.4:c.10379del in two Chinese AS families, which were located in exon 8, exon17, exon15, and exon16 of *ALMS1* gene respectively, including two frameshift pathogenic variants and two nonsense mutation. The correlation of pathogenic variants in exons 8 or 15, 16, 17 to phenotype remains to be determined. The sensorineural deafness typically occurs in nearly 84% of affected individuals with AS [15]. Interestingly, in this study sequencing of genomic DNA in two patients of family 2 revealed a compound heterozygote with two novel pathogenic variants in the ALMS1 gene, a new frameshift mutation NM_015120.4:c.10379del (NP_055935.4:p.(Asn3460IlefsTer49)) in exon 8 and a new nonsense mutation in NM_015120.4:c.10819C > T (NP_055935.4:p.(Arg3607Trp)) in exon 17, the clinical manifestation of the two patients did not have sensorineural deafness. Joy et al. [13], reported that a patient with AS did not have sensorineural deafness and sequencing of genomic DNA revealed 2 novel missense pathogenic variants in the ALMS1 gene in exon 17. Although no genotype phenotype correlations have been made for sensorineural deafness [15], it is possible that pathogenic variants in exon 17 may not be associated with sensorineural deafness. However serial studies based on large numbers of cases with these pathogenic variants are still required to confirm this possibility.

Alström Syndrome is considered a syndromic retinal ciliopathy. Sensory ciliopathy can impair an individual's perception of environmental cues, and in the outer segments of photoreceptors (part of the modified cilia) in the human retina, light interacts with photoreceptor proteins specialized for phototransduction, which, after a series of reactions, can eventually promote neurotransmitter release [3, 4]. Thus, as far as vision, cilia are where signal reception and initial transmission are made, followed by transmission of information to the cell body. Pathogenic variants in the ALMS1 protein may affect the transport of retinal and other proteins along photoreceptor axons, and transport dysfunction leads to a retinal dystrophy phenotype, as well as affects multi-organ cilia function. This is also a common feature of ciliopathies [5, 7].

The ocular phenotype of most of the previously reported cases was vision loss, nystagmus, and photophobia starting in early childhood. Most of them were clinically diagnosed as cone-rod dystrophy (CORD) according to existing fundus abnormality, including thinning of the retinal pigment epithelium, narrowing of the retinal blood vessels, extensive pigmentation, fluorescence imaging showing vascular filling, scattered patchy hyperfluorescence in the peripheral retina, OCT showing distortion of the outer retinal structures in the central macular recess, and autofluorescence showing a circular area of reduced self-fluorescence around the macula [11, 21, 17, 18]. Some were clinically diagnosed as LCA according to existing fundus abnormality, including optic disc pallor, macular pigment change, vascular stenosis, retinal pigment epithelium atrophy, granular appearance of the outer retina, extensive pigmentation of the fundus [17, 18]. The ocular phenotypes of the three affected children in two families of this study had ocular manifestations including poor visual acuity (VA), photophobia, nystagmus, color vision loss, mildly decline scotopic, and the severe reduced photopic response of ERG recording in early childhood, but no abnormal changes were found on the fundus. Therefore, the clinically diagnosis of three patients were made as achromatopsia initially. Although the clinical manifestations of AS are significantly, the first ocular symptoms are vision loss, nystagmus, photophobia, which may be the first reason for children visiting ophthalmology clinic, and provide an important clue for genetic screening and further clinical examinations to detect underlying diseases.

Moreover, the pathogenic variants in the three patients of two families in this study seem to have resulted in severe ocular phenotype and "mild-severity" systemic phenotype as judged by an early onset of cone dysfunction with low vision, total color blindness, obesity, sensorineural deafness (in proband of family 1), and mild abnormalities of lipid metabolism (in two patients of family 2), but lack of renal dysfunction, diabetes, elevation of TG levels and dilated cardiomyopathy. Due to genetic heterogeneity, the age of onset and the severity of clinical manifestations vary widely among individuals at the same mutated locus. There is no evidence of genotype–phenotype correlation, suggesting that unknown genetic or environmental factors may affect the phenotype. Mahamid et al.,[22] reported that a pair of brothers with AS had severe dilated cardiomyopathy since infancy. Still, the older brother's cardiomyopathy was cured as the disease progressed, while the younger brother's cardiac function deteriorated. In our study, three patients did not have cardiomyopathy.

Alström Syndrome is incurable currently, and most treatments are symptomatic. For obesity and hyperinsulinemia, strict diet control, strengthening exercise, and weight loss are given. Metformin hydrochloride tablets or insulin sensitizers such as pioglitazone can be taken orally to improve insulin resistance. If the patient is complicated with endocrine abnormalities such as hypothyroidism, hypogonadism, growth hormone deficiency, diabetes insipidus, and so on, complementary alternative therapies such as supplementation of levothyroxine sodium, testosterone, and growth hormone can be given [20, 15]. In this study, both families of the three affected children were informed that they should pay attention to the children's diet, strengthen exercise, regularly monitor blood glucose, lipids, and glycated hemoglobin, perform regular cardiac and renal ultrasound examinations, and pay attention to symptoms of other systemic systems for timely symptomatic treatment.

In conclusion, Alström Syndrome is a rare autosomal recessive cilia disease that causes retinal cone-roll dystrophy and systemic lesions due to the association of *ALMS1* protein with cilia function [23]. Thus, it is challenging to diagnose AS only by clinical examination. Genetic testing to identify the causative gene is necessary for accurate diagnosis and is currently the gold standard for AS diagnosis. Although Alström Syndrome is incurable now, early and comprehensive interventions can be made to prolong the patient's life and improve the quality of survival. Early diagnosis and life coaching can be helpful to slow down the patient's disease progress. Meanwhile, since the *ALMS1* gene causes AS, it is expected that with the development and application of gene testing and gene therapy and further development of science and technology such as embryo transplantation, systematic and practical treatments for this disease will come into being. Gene therapy and stem cell therapy are future research directions, and some studies have already confirmed the success of gene therapy techniques in restoring cilia function. As a result, they are expected to provide more treatments and effective therapeutic drugs.

## Data Availability

The datasets generated and analyzed during the current study are available in the [Banklt] repository (BankIt (https://www.ncbi.nlm.nih.gov/WebSub/) ID: 2,542,110).
